# Lipid Nanoparticles Potentiate CpG-Oligodeoxynucleotide-Based Vaccine for Influenza Virus

**DOI:** 10.3389/fimmu.2019.03018

**Published:** 2020-01-09

**Authors:** Seiki Shirai, Meito Shibuya, Atsushi Kawai, Shigeyuki Tamiya, Lisa Munakata, Daiki Omata, Ryo Suzuki, Taiki Aoshi, Yasuo Yoshioka

**Affiliations:** ^1^Laboratory of Nano-Design for Innovative Drug Development, Graduate School of Pharmaceutical Sciences, Osaka University, Osaka, Japan; ^2^Vaccine Creation Project, BIKEN Innovative Vaccine Research Alliance Laboratories, Research Institute for Microbial Diseases, Osaka University, Osaka, Japan; ^3^Laboratory of Drug and Gene Delivery Research, Faculty of Pharma-Science, Teikyo University, Tokyo, Japan; ^4^Vaccine Dynamics Project, BIKEN Innovative Vaccine Research Alliance Laboratories, Research Institute for Microbial Diseases, Osaka University, Osaka, Japan; ^5^BIKEN Center for Innovative Vaccine Research and Development, The Research Foundation for Microbial Diseases of Osaka University, Osaka, Japan; ^6^Global Center for Medical Engineering and Informatics, Osaka University, Osaka, Japan

**Keywords:** adjuvant, CpG oligodeoxynucleotide, influenza virus, interferon-α, lipid nanoparticle, vaccine

## Abstract

Current influenza vaccines are generally effective against highly similar (homologous) strains, but their effectiveness decreases markedly against antigenically mismatched (heterologous) strains. One way of developing a universal influenza vaccine with a broader spectrum of protection is to use appropriate vaccine adjuvants to improve a vaccine's effectiveness and change its immune properties. Oligodeoxynucleotides (ODNs) with unmethylated cytosine-phosphate-guanine (CpG) motifs (CpG ODNs), which are Toll-like-receptor 9 (TLR9) agonists, are among the most promising adjuvants and are already being used in humans. However, the development of novel delivery vehicles to improve adjuvant effects *in vivo* is highly desirable. Here, we assessed the potential of lipid nanoparticles (LNPs) as CpG ODN delivery vehicles in mice to augment the vaccine adjuvant effects of CpG ODN and enhance the protective spectrum of conventional influenza split vaccine (SV). *In vitro*, compared with CpG ODN, LNPs containing CpG ODNs (LNP-CpGs) induced significantly greater production of cytokines such as IL-12 p40 and IFN-α by mouse dendritic cells (DCs) and significantly greater expression of the co-stimulatory molecules CD80 and CD86 on DCs. In addition, after subcutaneous administration in mice, compared with CpG ODN, LNP-CpGs enhanced the expression of CD80 and CD86 on plasmacytoid DCs in draining lymph nodes. LNP-CpGs given with SV from H1N1 influenza A virus improved T-cell responses and gave a stronger not only SV-specific but also heterologous-virus-strain-specific IgG2c response than CpG ODN. Furthermore, immunization with SV plus LNP-CpGs protected against not only homologous strain challenge but also heterologous and heterosubtypic strain challenge, whereas immunization with SV plus CpG ODNs protected against homologous strain challenge only. We therefore demonstrated that LNP-CpGs improved the adjuvant effects of CpG ODN and broadened the protective spectrum of SV against influenza virus. We expect that this strategy will be useful in developing adjuvant delivery vehicles and universal influenza vaccines.

## Introduction

Influenza viruses are of serious public health concern: annual epidemics caused by these viruses affect 5–15% of the global population and induce ~3–5 million cases of severe illness (1). One of the most critical strategies for preventing the spread of influenza viruses at the population level is vaccination. However, current influenza vaccines are generally effective against highly similar (homologous) virus strains, but their effectiveness decreases markedly against antigenically mismatched (heterologous) strains (1, 2). Because influenza viruses undergo constant genetic and antigenic changes owing to the high rate of point mutations within the influenza virus genome, mismatch between vaccine strains and seasonal circulating viruses is not fully avoided and still occurs frequently (3, 4). Clearly, we need to develop a next generation of universal influenza vaccines that give a broader spectrum of protection against seasonal influenza viruses.

Virus-specific IgG2 antibodies play a predominant protective role in the response to influenza virus infection or vaccination against influenza viruses (5). In fact, the marked IgG1 to IgG2c shift in influenza-virus-specific antibodies upon vaccination by using an adjuvant augments protection against heterologous virus challenge (6), suggesting that one way of improving the effectiveness of influenza vaccines against heterologous viruses is to change the virus-specific antibody isotype from IgG1 to IgG2 by using appropriate vaccine adjuvants. Nowadays, alum (aluminum salts) is one of the vaccine adjuvants most widely used in many important vaccines in humans (7). Unfortunately, however, alum cannot induce Th1-type immune responses and antigen-specific IgG2 production, although it can induce strong Th2-type immune responses and antigen-specific IgG1 production (7). In contrast, oligodeoxynucleotides (ODNs) with unmethylated cytosine-phosphate-guanine (CpG) motifs (CpG ODNs) are appropriate adjuvants for inducing Th1-type immune responses and antigen-specific IgG2 production (8, 9). A CpG ODN is a short single-stranded synthetic DNA fragment containing the immunostimulatory CpG motif and binding to Toll-like receptor 9 (TLR9) at the endosomes after uptake by dendritic cells (DCs). TLR9 primarily binds unmethylated CpG DNA motifs, which are common in bacterial and viral DNA, and it plays a central role in viral immunity as well as various autoimmune disorders (10). TLR9 activates bifurcated signals downstream of MyD88 to induce the upregulation of pro-inflammatory cytokine and type I interferon (IFN) genes in macrophages, DCs, and B cells (10). There are several types of CpG ODNs, each of which has a different structure, physical properties, and immunostimulatory properties (9). Among them, D-type CpG ODNs (also known as A-type CpG ODNs) activate plasmacytoid DCs (pDCs) to produce massive amounts of type I IFNs such as IFN-α, which has crucial roles in the activation and cytotoxicity of natural killer T cells, the activation of CD8^+^ T cells, and the maturation of DCs (11, 12). D-type CpG ODNs have shown potential in preclinical and clinical studies as adjuvants in vaccines for infectious diseases, cancers, and allergic asthma (13–17). However, CpG ODNs—especially D-type CpG ODNs—are generally prone to degradation by nucleases such as DNase *in vivo*, resulting in a decrease in adjuvant activity, because D-type CpG ODNs have a naturally occurring phosphodiester backbone (8, 9, 14). To overcome this problem and enhance the adjuvant activity of CpG ODNs by their efficient delivery to DCs, a delivery vehicle must be developed to protect the CpG ODNs from degradation by nucleases.

Several functionalized nanoparticles, including polymer- and lipid-based particles, have been employed as delivery vehicles for DNA- or RNA-based medicines. Each vehicle has merits and demerits (18–20). Lipid nanoparticles (LNPs), which are typically composed of an ionizable lipid, cholesterol, lipid conjugated with polyethylene glycol (PEGylated lipid), and a helper lipid, have recently attracted much attention as novel lipid-based delivery vehicles for DNA- or RNA-based medicines (19, 21). LNPs can be readily produced with highly efficient encapsulation of DNA- or RNA-based medicines by using a microfluidic mixer (22–24). In addition, previous reports have shown the safety of LNPs in humans after intravenous administration (25). LNPs facilitate the delivery of short interfering RNA (siRNA) and mRNA to several types of cells, including immune cells, as well as the release of these oligonucleotides from phagosomes or endosomes into the cytoplasm. For example, siRNA- or mRNA-loaded LNPs have been used for gene therapy of hepatic diseases and for cell-specific delivery of mRNA (26–28). Furthermore, LNPs are expected to be useful as delivery vehicles for vaccines. For example, LNPs containing mRNA coding for protein antigens have been used to induce antigen-specific immune responses and protect against Zika virus, influenza virus, and cytomegalovirus in mice and rhesus macaques (29–34). In the vaccine field, LNPs are expected to be used as delivery vehicles for not only mRNA but also peptide or protein antigens and adjuvants. Thus, LNPs might have potential as CpG ODN-delivery vehicles. Our group recently reported the usefulness of LNPs containing CpG ODN as immunostimulatory drugs for cancer immunotherapy (35). We showed that both intratumoral and intravenous administration of LNPs containing CpG ODN enhanced the effect of CD8^+^ T cells in reducing tumor growth in mice (35). However, the usefulness of LNPs containing CpG ODNs as vaccine adjuvants remains unclear.

Here, we show the usefulness of LNPs as CpG ODN-delivery vehicles to improve both cytokine production by DCs and the expression of co-stimulatory molecules on DCs *in vitro* and *in vivo*. In addition, LNPs containing CpG ODN and given with influenza split vaccine (SV) provoked a stronger antigen-specific IgG2 response than CpG ODN, and they gave superior cross-protection against heterologous influenza virus challenge. These data thus demonstrate the usefulness of LNPs for improving the adjuvant activity of CpG ODN and broadening the protective spectrum of SVs for influenza virus.

## Materials and Methods

### Reagents

1,2-dioleoyl-3-trimethylammonium-propane was purchased from Lipoid GmbH (Ludwigshafen, Germany). 1,2-dipalmitoyl-sn-glycero-3-phosphocholine and N-(carbonyl-methoxypolyethyleneglycol 2000)-1,2-distearoyl-sn-glycero-3-phosphoethanolamine were purchased from NOF Corporation (Tokyo, Japan). Cholesterol was purchased from Fujifilm Wako Pure Chemical Corporation, Ltd. (Osaka, Japan). D-type CpG ODN (CpG ODN: 5′-ggtgcatcgatgcagggggg-3′) was purchased from GeneDesign (Osaka, Japan). Horseradish-peroxidase-conjugated goat anti-mouse IgG was purchased from Merck Millipore (Darmstadt, Germany). Horseradish-peroxidase-conjugated goat anti-mouse IgG1 and IgG2c were purchased from SouthernBiotech (Birmingham, AL, USA). Alum was purchased from InvivoGen (San Diego, CA, USA). Ether-treated hemagglutinin-antigen-enriched virion-free SV from H1N1 influenza A virus [strain: A/California/7/2009 (Cal7)], SV from H3N2 influenza A virus (strain: A/Texas/50/2012 [Tex50]) and H1N1 influenza A virus (strain: A/Puerto Rico/8/34 [PR8]) were kindly provided by Dr. Yasuyuki Gomi of the Research Foundation for Microbial Diseases, Osaka University, Japan. H1N1 influenza A virus (strain: Cal7) was kindly provided by Dr. Hideki Asanuma of the National Institute of Infectious Diseases, Japan.

### Mice

C57BL/6J mice were purchased from SLC (Hamamatsu, Japan). Mice were housed in a room with a 12:12-h light:dark cycle (lights on, 8:00 a.m.; lights off, 8:00 p.m.) and had unrestricted access to food and water. All animal experiments were performed in accordance with Osaka University's institutional guidelines for the ethical treatment of animals (protocol number H26-11-0).

### Synthesis of LNP-CpGs

Two types of LNPs containing CpG ODN (LNP-CpGs) were prepared by using NanoAssemblr Benchtop (Precision NanoSystems Inc., BC, Canada), which can mediate bottom-up self-assembly for nanoparticle synthesis with microfluidic mixing technology. Briefly, 1,2-dioleoyl-3-trimethylammonium-propane, 1,2-dipalmitoyl-sn-glycero-3-phosphocholine, cholesterol, and N-(carbonyl-methoxypolyethyleneglycol 2000)-1,2-distearoyl-sn-glycero-3-phosphoethanolamine were, respectively, dissolved in ethanol at a molar ratio of 50: 19.5: 30: 0.5 or 50: 17: 30: 3. CpG ODN was prepared in 25 mM acetate buffer at pH 4.0. The lipid solution (10 mg/mL) in ethanol and CpG ODN solution were, respectively, injected into the microfluidic mixer at a volumetric ratio of 1:3 and combined at final flow rate of 15 mL/min (3.75 mL/min ethanol, 11.25 mL/ min aqueous). The mixtures were immediately dialyzed (50-kD MWCO dialysis tubing, Repligen Corporation, Waltham, MA, USA) against 5% glucose solution to remove the ethanol and unloaded CpG ODN. Each prepared LNP-CpG was then concentrated to ~0.7 mg/mL CpG ODN by using Amicon Ultra centrifugal filters (100-kD MWCO, Merck KGaA, Darmstadt, Germany) and filtered through a 0.22-μm PVDF filter (Merck KGaA). The theoretical CpG-ODN-to-lipid ratios for all formulations were maintained at an N/P charge ratio (the ratio of the charge on the cationic lipid, assuming that it was in the positively charged protonated form, to the negative charge on the CpG ODN) of 3. All LNP preparation work was performed at room temperature. We used 5% glucose solution as a control buffer in all experiments, because 5% glucose solution was used for the LNP-CpGs.

### Analysis of Lipid Nanoparticles

The size distributions of the two types of LNP-CpG were measured by using dynamic light scattering (Zetasizer Nano-ZS, Malvern Panalytical Ltd., Malvern, UK). The CpG ODN concentration in the LNP-CpGs was measured with PicoGreen reagent (Quant-iT PicoGreen dsDNA Assay Kit, Thermo Fisher Scientific, San Jose, CA, USA). Briefly, LNP-CpG was incubated at 37°C for 10 min in the presence of 1% Triton X-100 (Wako Pure Chemical Industries) and PicoGreen reagent was added. The fluorescence intensity (excitation/emission wavelength, 485/528 nm) was then measured.

### Production and Purification of Recombinant Hemagglutinin (HA) and Neuraminidase (NA) Proteins

The amino acid sequences for the HA used here were derived from Cal7 (GenBank accession number: ACV82259.1) and PR8 (GenBank accession number: LC120393.1). The amino acid sequences for the NA that we used were derived from Cal7 (GenBank accession number: MN596847.1). Human codon-optimized cDNA of the ectodomain of HA with a C-terminal histidine tag (His-tag) and of NA with an N-terminal His-tag was cloned into a pcDNA3.1 expression plasmid (Thermo Fisher Scientific). The codon-optimized fold on trimerization domain sequence and tetrabrachion tetramerization domain sequence were inserted at the C-terminal of HA and the N-terminal of NA, respectively, as previously described (36, 37). All secreted soluble recombinant HAs and recombinant NA were expressed by using the Expi293 Expression System (Thermo Fisher Scientific), in accordance with the manufacturer's instructions. The recombinant HAs and recombinant NA in the supernatant were then purified by using an AKTAexplorer chromatography system with an Ni-Sepharose HisTrap FF column (GE Healthcare, Diegem, Belgium) and a Superose 6 Increase 10/300 GL column (GE Healthcare).

### Preparation and Stimulation of Mouse Bone-Marrow-Derived DCs

To generate bone-marrow-derived DCs, we isolated bone marrow cells from the femurs of C57BL/6J mice and cultured the cells at 37°C for 7 days with 100 ng/mL human Fms-related tyrosine kinase 3 ligand (PeproTech, Rocky Hill, NJ, USA). Cells were seeded at a density of 1 × 10^5^ cells/well in a 96-well flat-bottomed culture plate (Nunc, Roskilde, Denmark) and were cultured in complete RPMI medium (RPMI 1640 supplemented with 10 vol. % fetal calf serum, penicillin, and streptomycin). These cells were stimulated with CpG ODN or with each LNP-CpG for 24 h. Supernatants were subjected to ELISA to determine the levels of IFN-α (InvivoGen) and interleukin (IL)-12 p40 (BioLegend, San Diego, CA, USA), in accordance with the manufacturers' instructions. To check the levels of co-stimulatory molecules on DCs, we incubated the cells with anti-mouse CD16/CD32 antibody (BioLegend), anti-CD11c antibody (BioLegend), anti-CD11b antibody (BioLegend), anti-CD80 antibody (BioLegend), or anti-CD86 antibody (BioLegend). Then the cells were analyzed by means of flow cytometry (NovoCyte Flow Cytometer, ACEA Biosciences, San Diego, CA, USA).

### Expression Levels of Co-stimulatory Molecules on DCs in Draining Lymph Nodes

C57BL/6J mice were treated with CpG ODN (10 μg/mouse) or each LNP-CpG (10 μg CpG ODN/mouse) subcutaneously at the base of the tail. Twenty-four hours after administration, the lymph nodes draining the site of administration were collected after euthanasia. To prepare single-cell suspensions, the draining lymph nodes were incubated with 200 μg/mL Liberase TL (Roche Diagnostics GmbH, Mannheim, Germany) and 10 U/mL DNase I (Roche Diagnostics GmbH) for 60 min at 37°C. Prepared cells were incubated with anti-mouse CD3ε antibody (BioLegend), anti-mouse CD19 antibody (BioLegend), anti-mouse CD16/CD32 antibody, anti-CD11c antibody, anti-PDCA-1 antibody (BioLegend), anti-CD80 antibody, or anti-CD86 antibody for flow cytometry. In this way, the DCs were separated into two subsets, namely CD3ε^−^ CD19^−^ PDCA-1^+^ CD11c^+^ pDCs and CD3ε^−^ CD19^−^ PDCA-1^−^ CD11c^+^ conventional DCs (cDCs). The cells were then analyzed by means of flow cytometry.

### Cytokine Production by Splenocytes After Immunization

C57BL/6J mice were treated with SV from Cal7 (0.5 μg/mouse) without or with CpG ODNs (10 μg/mouse), each LNP-CpG (CpG ODN 10 μg/mouse), or alum (50 μg/mouse) subcutaneously at the base of the tail on days 0 and 21. On day 28, spleens were collected and splenocytes were prepared for determining IFN-γ production. Splenocytes (1 × 10^6^ cells) were added to the wells of a 96-well plate. They were then stimulated with SV (final concentration, 10 μg/mL) for 1 or 5 days at 37°C or left unstimulated. After the incubation, the concentrations of IL-2, IL-13, and IFN-γ in the supernatants were analyzed by ELISA (IL-2 and IFN-γ: BioLegend; IL-13: eBioscience, San Diego, CA, USA) in accordance with the manufacturers' instructions.

### Vaccine Against Influenza Virus

C57BL/6J mice were immunized subcutaneously at the base of the tail on days 0 and 21 by using SV from Cal7 (0.5 μg/mouse) or SV from Tex50 (0.5 μg/mouse) without or with CpG ODNs (10 μg/mouse), each LNP-CpG (CpG ODN 10 μg/mouse), or alum (50 μg/mouse). On day 28, we obtained plasma samples, and the levels of SV- or influenza-A-virus (PR8)-specific antibodies in the plasma were determined by ELISA. To detect SV- or PR8-specific IgG, IgG1, and IgG2c, ELISA plates (Corning, Corning, NY, USA) were coated with SV (10 μg/mL) or influenza A virus (PR8: 1 μg/mL) in phosphate-buffered saline (PBS) overnight at 4°C. The coated plates were then incubated with 1% Block Ace for 2 h at room temperature (DS Pharma Biomedical, Osaka, Japan). Plasma samples were diluted with 0.4% Block Ace, and these dilutions were added to the antigen-coated plates. After incubation with plasma for 2 h at room temperature, the coated plates were incubated with a horseradish-peroxidase-conjugated goat anti-mouse IgG, IgG1, or IgG2c solution for 1 h at room temperature. After the incubation, the color reaction was developed with tetramethyl benzidine (Nacalai Tesque, Kyoto, Japan), stopped with 2 N H_2_SO_4_, and measured at OD_450−570_ on a microplate reader (Power Wave HT, BioTek, Winooski, VT, USA). On day 31, mice were challenged intranasally with 3 × 10^4^ TCID_50_ of Cal7 or 1.2 × 10^3^ TCID_50_ of PR8 in 30 μL of PBS under anesthesia (38). Body weights and survival rates of challenged mice were monitored. The humane endpoint was set at 25% bodyweight loss relative to the initial body weight at the time of infection.

### Neutralization Assay

Plasma samples were incubated with RDE (Receptor Destroying Enzyme) (II) (Denka Seiken, Tokyo, Japan) for 18 h at 37°C and then heated at 56°C for 1 h to deactivate the enzyme. Mixtures of two-fold serial-diluted plasma samples and influenza virus with final concentrations of 100 × TCID_50_ virus per mixture were incubated at 37°C for 30 min. After being washed with PBS, mixtures were subsequently added to Madin-Darby canine kidney (MDCK) cells and incubated at 37°C for 3 days. The cells were fixed with 4% paraformaldehyde at room temperature for 10 min. The cells were stained with 0.1% amido black (Nacalai Tesque) in acetic acid solution at room temperature for 30 min. After the cells had been washed with water, 0.1 N NaOH was added and the OD_630_ was measured on a microplate reader (Power Wave HT, BioTek).

### Recovery of Bronchoalveolar Lavage Fluid (BALF)

On day 5 after influenza virus challenge, mice were euthanized under anesthesia. BALF was obtained by lavaging the lung with 1 mL of PBS. The BALF was centrifuged at 600 × g for 5 min, and the supernatant was used to measure virus titers. Virus titers were assessed by infection of MDCK cells as described above.

### Statistical Analyses

Statistical analyses were performed with Prism (GraphPad Software, San Diego, CA, USA). All data are presented as means with standard deviation (SD). Significant differences were determined by means of Tukey's test. Significant differences in survival rates were obtained by comparing Kaplan–Meier curves by using the log-rank test. A *P* value of <0.05 was considered to indicate statistical significance.

## Results

### LNP-CpGs Improve Cytokine Production and Co-stimulatory Molecule Expression on Mouse DCs

We investigated the usefulness of LNPs as CpG ODN delivery vehicles. LNP-CpGs were prepared by using NanoAssemblr, a microfluidic mixer system. We constructed two types of LNP-CpG with different amounts of polyethylene glycol (PEG)-conjugated lipid, 0.5 or 3 mol % [LNP(0.5%)-CpG or LNP(3%)-CpG, respectively]; we had already confirmed the physical properties of each LNP-CpG (35). The size distribution spectrum of each LNP-CpG showed essentially a single peak, indicating that these particles had a narrow size range and were monodispersed. The hydrodynamic diameter of LNP(0.5%)-CpG was 54.0 nm (polydispersity index: 0.157) and of LNP(3%)-CpG was 43.0 nm (polydispersity index: 0.089). The zeta potentials of LNP(0.5%)-CpG and LNP(3%)-CpG were 1.12 ± 8.26 and 0.34 ± 11.2 mV, respectively. In addition, transmission electron microscopy showed that both LNP-CpGs were spherical with a fully filled packed core, indicating that both LNP-CpGs formed lipid nanoparticles (35).

**Figure 1:**
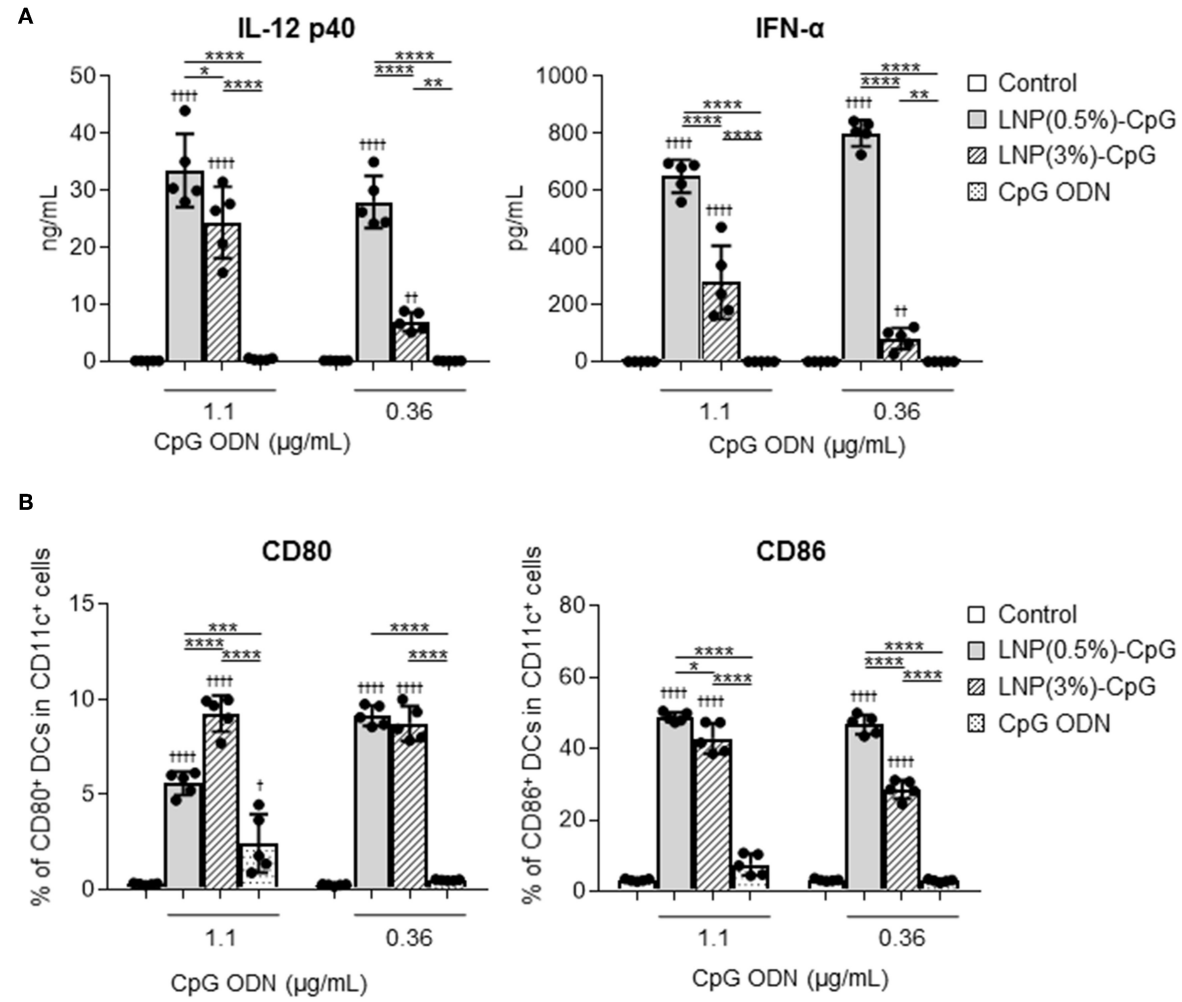
Activation of mouse-derived dendritic cells (DCs) by LNP-CpGs *in vitro*. Mouse-derived DCs were treated with CpG ODN or with each LNP-CpG for 24 h *in vitro*. The CpG ODN content was the same between the cells treated with CpG ODN alone and those treated with LNP-CpGs. **(A)** Cytokine production. Levels of IL-12 p40 and IFN-α in the supernatants were measured by ELISA. **(B)** Expression of co-stimulatory molecules. Expression of CD80 and CD86 on DCs was measured by flow cytometry; percentages of positive DCs are shown. **(A,B)** *n* = 5 per group. Data are means ± SD. ^†^*P* < 0.05, ^††^*P* < 0.01, ^††††^*P* < 0.0001 vs. untreated control group; *P < 0.05, **P < 0.01, ***P < 0.001, ****P < 0.0001 as indicated by Tukey's test.

To determine the immune-stimulatory activity of LNP-CpGs on DCs, mouse-derived DCs were treated with CpG ODN or with each LNP-CpG *in vitro*, and we examined the levels of cytokines in the supernatants (Figure 1A) and the expression levels of co-stimulatory molecules on DCs (Figure 1B). DCs stimulated with each LNP-CpG produced significantly higher levels of IL-12 p40 and IFN-α than those treated with CpG ODN at the same CpG ODN concentration (Figure 1A). Cytokine levels in the LNP(0.5%)-CpG-treated group were significantly higher than those in the LNP(3%)-CpG-treated group (Figure 1A). In addition, DCs stimulated with each LNP-CpG expressed significantly more CD80 and CD86 than those treated with CpG ODN alone, and LNP(0.5%)-CpG induced significantly greater expression of CD86 on DCs than did LNP(3%)-CpG (Figure 1B). These data suggested that LNP-CpGs had superior immune-stimulatory activity to CpG ODN *in vitro*.

### LNP-CpG Enhances the Expression of Co-stimulatory Molecules on pDCs *in vivo*

**Figure 2:**
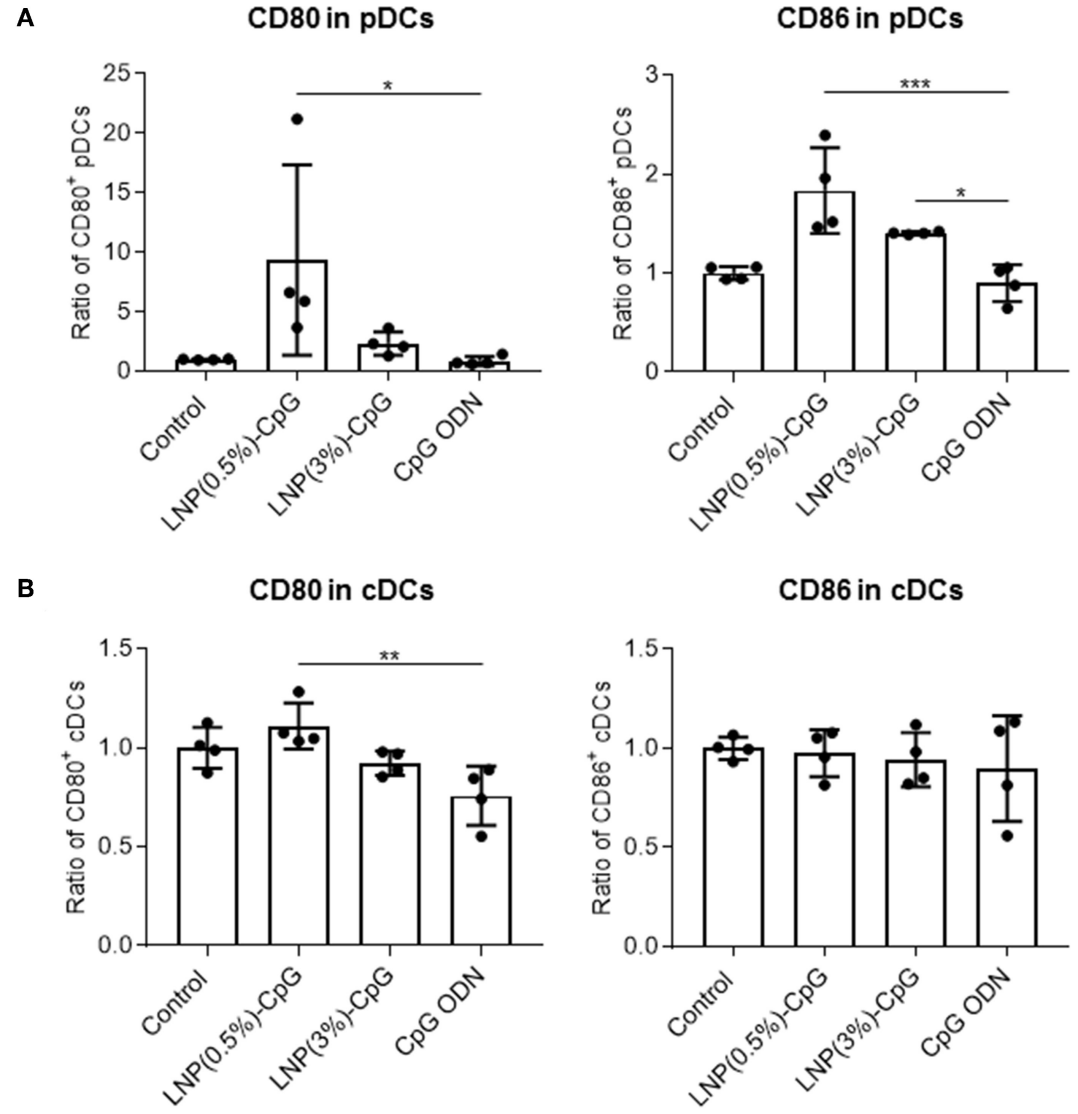
Activation of plasmacytoid dendritic cells (pDCs) by LNP-CpGs *in vivo*. CpG ODN (10 μg per mouse) or each LNP-CpG (10 μg CpG ODNs per mouse) was administered to mice subcutaneously. After 24 h, cells in the draining lymph nodes were harvested, and the expression of CD80 and CD86 on pDCs **(A)** and conventional dendritic cells (cDCs) **(B)** was measured by flow cytometry. DCs were separated into PDCA-1^+^ CD11c^+^ pDCs and PDCA-1^−^ CD11c^+^ cDCs. Mean fluorescence intensity ratios obtained for the samples relative to the control group are shown. **(A,B)** *n* = 4 per group. Data are means ± SD. *P < 0.05, **P < 0.01, ***P < 0.001 as indicated by Tukey's test.

To determine the immune-stimulatory activity of LNP-CpGs *in vivo*, we analyzed the expression levels of CD80 and CD86 on DCs in the draining lymph nodes after subcutaneous administration (Figure 2). We categorized the DCs into pDCs (PDCA1^+^ CD11c^+^) and cDCs (PDCA1^−^ CD11c^+^). LNP(0.5%)-CpG administration resulted in significantly greater expression levels of CD80 and CD86 on pDCs than did CpG ODN, whereas CpG ODN did not enhance the expression of these molecules compared with the control (Figure 2A). The expression level of CD86 on pDCs in the LNP(3%)-CpG-treated group was significantly higher than that in the CpG-ODN-treated group (Figure 2A). In cDCs, the expression level of CD80 in the LNP(0.5%)-CpG-treated group was significantly higher than that in the CpG-ODN-treated group, although the relative increase was small (Figure 2B). These data suggest that LNP-CpGs enhanced immune-stimulatory activity relative to CpG ODN, both *in vitro* and *in vivo*.

### LNP-CpG Improves T-Cells Responses and Antigen-Specific Antibody Responses in an Influenza Vaccine (Cal7 SV) Model

**Figure 3:**
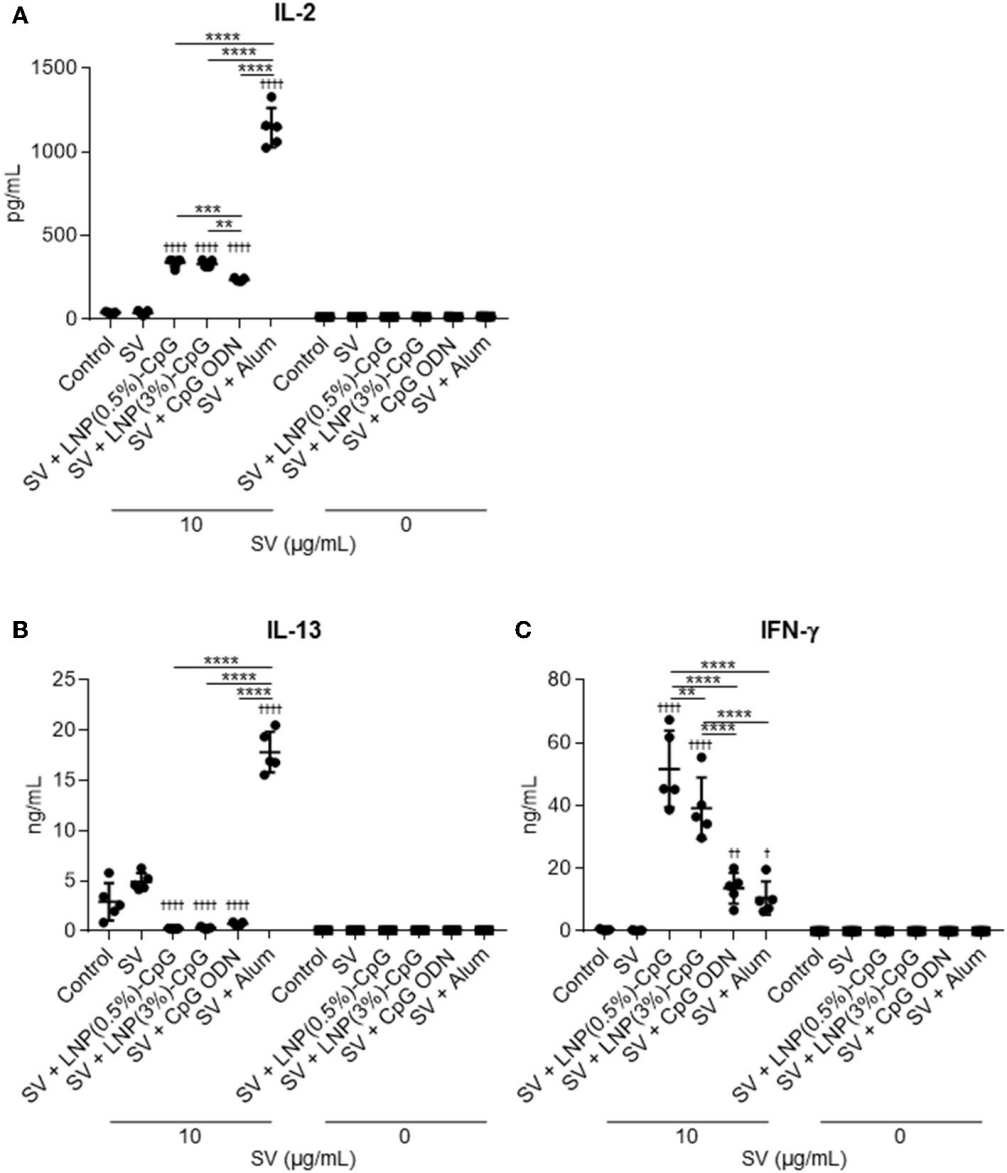
Influenza-virus-specific T-cell responses *in vivo*. Mice were immunized subcutaneously with split vaccine (SV) alone, SV plus CpG ODN, or SV plus either LNP-CpG. After the last immunization, splenocytes were cultured in the presence or absence of SV *in vitro*. After 1 (for IL-2) or 5 (for IL-13 and IFN-γ) days, the levels of IL-2 **(A)**, IL-13 **(B)**, and IFN-γ **(C)** were measured by using ELISA. **(A–C)** *n* = 5 per group. Data are means ± SD. ^†^*P* < 0.05, ^††^*P* < 0.01, ^††††^*P* < 0.0001 vs. group immunized with SV alone; **P < 0.01, ***P < 0.001, ****P < 0.0001 as indicated by Tukey's test.

We examined the vaccine-adjuvant effect of LNP-CpGs in a clinically relevant influenza vaccination model in mice. We used conventional seasonal SV from Cal7 as an antigen. Mice were immunized with SV plus either LNP-CpG (10 μg CpG ODN/mouse), or with SV plus CpG ODN (10 μg/mouse). We used alum as a positive control for the adjuvant. First, to investigate T-cell responses, splenocytes were recovered from the spleen after immunization and stimulated with SV *in vitro*, and the levels of IL-2, IL-13, and IFN-γ in the supernatant were measured by using an ELISA (Figure 3). The levels of IL-2 and IL-13 in mice immunized with SV plus alum were significantly higher than those in mice given SV alone, or SV plus CpG ODN, or SV plus either LNP-CpG (Figures 3A,B). The level of IL-2 in mice given SV plus either LNP-CpG was significantly higher than that in mice given SV plus CpG ODN (Figure 3A). In addition, the levels of IFN-γ were significantly higher in mice immunized with SV plus either LNP-CpG than in those given SV alone, or SV plus CpG ODN, or SV plus alum (Figure 3C). These results suggested that LNP-CpG improved Th1 responses compared with CpG ODN.

**Figure 4:**
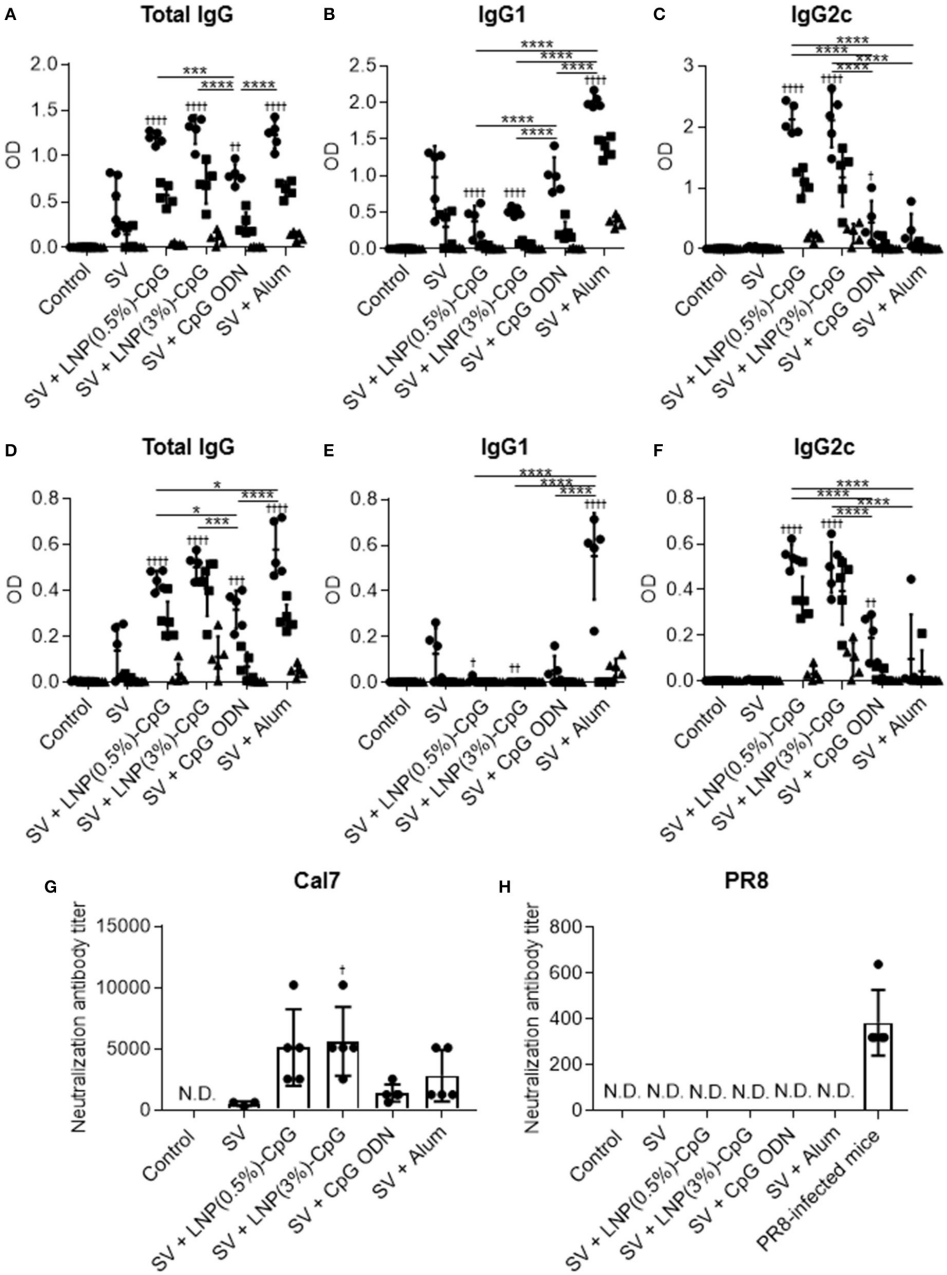
Influenza-virus-specific antibody responses *in vivo*. Mice were immunized subcutaneously with split vaccine (SV) alone, SV plus CpG ODN, SV plus either LNP-CpG, or SV plus alum. **(A–F)** Antibody responses. Levels of SV-specific total IgG **(A)**, IgG1 **(B)**, and IgG2c **(C)**, and of PR8-specific total IgG **(D)**, IgG1 **(E)**, and IgG2c **(F)** in plasma were evaluated by using ELISA 7 days after final immunization. We used 6000- (•), 30,000- (■), and 150,000- (▴) fold diluted plasma samples. **(G,H)** Neutralization titers against influenza virus. Neutralization titers in plasma samples against Cal7 **(G)** and PR8 **(H)** were evaluated. **(A–F)** *n* = 5 per group. Data are means ± SD. Significant differences were analyzed only in the 6,000-fold-diluted plasma samples. ^†^*P* < 0.05, ^†††^*P* < 0.001, ^††††^*P* < 0.0001 vs. group immunized with SV alone; *P < 0.05, ***P < 0.001, ****P < 0.0001 as indicated by Tukey's test. **(G,H)** N.D., not detected. *n* = 3 (SV alone) or 5 per group. Data are means ± SD. ^†^*P* < 0.05 vs. group immunized with SV alone.

Next, the plasma levels of SV-specific total IgG (Figure 4A), IgG1 (Figure 4B), and IgG2c (Figure 4C) antibodies were analyzed by using ELISA after last immunization. Mice immunized with SV plus LNP-CpGs produced significantly higher levels of SV-specific total IgG than those given SV plus CpG ODN, whereas SV plus CpG ODN induced significantly greater levels of SV-specific total IgG than did SV alone (Figure 4A). There were no significant differences in SV-specific total IgG levels among mice immunized with SV plus either LNP-CpG and those given SV plus alum (Figure 4A). Furthermore, the level of SV-specific IgG2c in mice immunized with SV plus either LNP-CpG was significantly higher than that in mice immunized with SV plus CpG ODN or SV plus alum (Figure 4C). In contrast, the level of SV-specific IgG1 in mice immunized with SV plus either LNP-CpG was significantly lower than that in mice immunized with SV plus CpG ODN or SV plus alum (Figure 4B). Mice immunized with SV plus alum had the highest level of SV-specific IgG1 among all groups (Figure 4B).

Next, to compare cross-protective effects after vaccination, we examined the plasma levels of total IgG antibodies specific to PR8, an influenza virus heterologous to the one used as a vaccine (Figure 4D), as well as the levels of IgG1 (Figure 4E) and IgG2c (Figure 4F) specific to this virus, by using the same plasma samples as used to assess antibodies to SV. Consistent with the SV-specific antibody responses, the level of PR8-specific total IgG in mice given SV plus either LNP-CpG was significantly higher than that in mice given SV plus CpG ODN (Figure 4D). In addition, SV plus alum induced significantly greater levels of PR8-specific total IgG than did LNP(0.5%)-CpG (Figure 4D). SV plus either LNP-CpG induced significantly higher PR8-specific IgG2c levels than did SV plus CpG ODN or SV plus alum (Figure 4F), whereas SV plus alum induced the highest PR8-specific IgG1 levels among all groups (Figure 4E). These results suggested that LNP-CpGs enhanced not only homologous- but also heterologous-virus-specific total IgG and IgG2 compared with CpG ODN.

Neutralization assays are generally used to evaluate the neutralizing activity of antibodies against influenza virus. Next, we examined the neutralizing activity of plasma from immunized mice against Cal7 (Figure 4G) and PR8 (Figure 4H). The neutralizing activity against Cal7 showed the same trend as the levels of SV-specific total IgG in Figure 4A: plasma from mice immunized with SV plus either LNP-CpG or with SV plus alum had greater neutralizing activity than those given SV plus CpG ODN (Figure 4G). In contrast, we did not observe any neutralizing activity against PR8 among any of the plasma samples, although the plasma samples from PR8-infected mice, which served as positive controls, had high neutralizing activity (Figure 4H). These results suggested that antibodies induced by SV plus LNP-CpGs could not neutralize infection with the heterologous strain, although these antibodies could bind to this strain.

**Figure 5:**
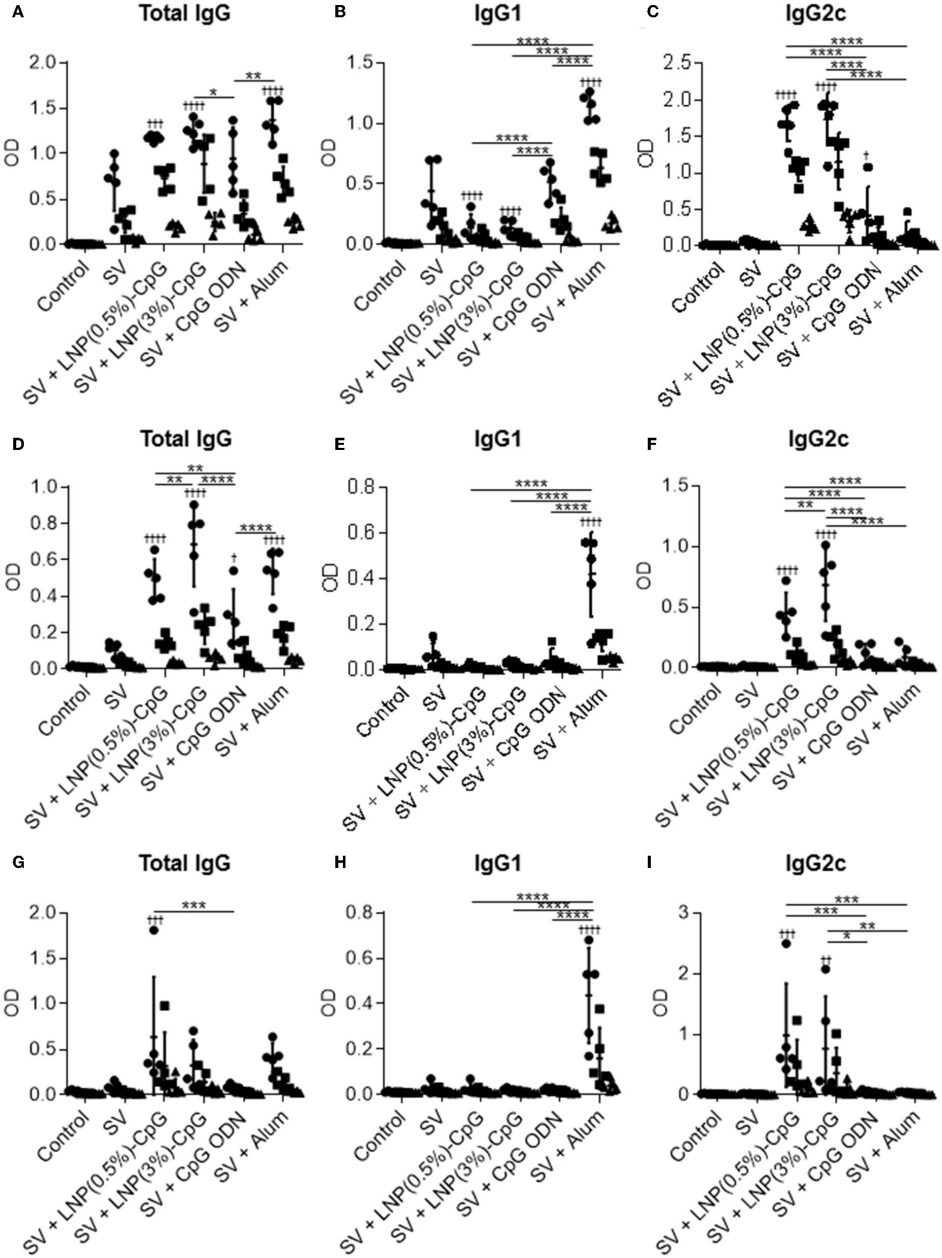
Hemagglutinin (HA)- and neuraminidase (NA)-specific antibody responses *in vivo*. Levels of HA from Cal7-specific total IgG **(A)**, IgG1 **(B)**, and IgG2c **(C)**; of NA from Cal7-specific total IgG **(D)**, IgG1 **(E)**, and IgG2c **(F)**; and of HA from PR8-specific total IgG **(G)**, IgG1 **(H)**, and IgG2c **(I)** in plasma were evaluated by using ELISA 7 days after final immunization. The same plasma samples as used in Figure 4 were used here. We used 800- (•), 4000- (■), and 20,000- (▴) fold diluted plasma samples for **(A–F)** and 32- (•), 160- (■), and 800- (▴) fold diluted plasma samples for **(G–I)**. *n* = 5. Data are means ± SD. Significant differences were analyzed only in the 800-fold-diluted plasma samples **(A–F)** and the 32-fold-diluted plasma samples **(G–I)**. ^†^*P* < 0.05, ^††^*P* < 0.01, ^†††^*P* < 0.001, ^††††^*P* < 0.0001 vs. group immunized with SV alone; *P < 0.05, **P < 0.01, ***P < 0.001, ****P < 0.0001 as indicated by Tukey's test.

To examine the antigen specificity of plasma from immunized mice, after the last immunization we used ELISA to examine the plasma levels of total IgG (Figures 5A,D,G), IgG1 (Figures 5B,E,H), and IgG2c (Figures 5C,F,I) antibodies specific to recombinant HA from Cal7 (Figures 5A–C), recombinant NA from Cal7 (Figures 5D–F), and recombinant HA from PR8 (Figures 5G–I). Consistent with the SV-specific antibody responses, the levels of HA- and NA-specific IgG2c in mice given SV plus either LNP-CpG were significantly higher than those in mice given SV plus CpG ODN (Figures 5C,F,I). Both LNP-CpGs induced not only IgG2c specific to HA from Cal7 (Figure 5C) but also IgG2c specific to HA from PR8 (Figure 5I), although the levels of IgG2c specific to HA from PR8 were lower than those of IgG2c specific to HA from Cal7. In contrast, the level of HA- and NA-specific IgG1 in mice immunized with SV plus either LNP-CpG was significantly lower than that in mice immunized with SV plus alum (Figures 5B,E,H). These results suggest that both LNP-CpGs enhanced not only homologous HA- and NA- but also heterologous-HA-specific IgG2.

### LNP-CpGs Have Strong Preventive Effects Against Heterologous Influenza Virus Challenge

**Figure 6:**
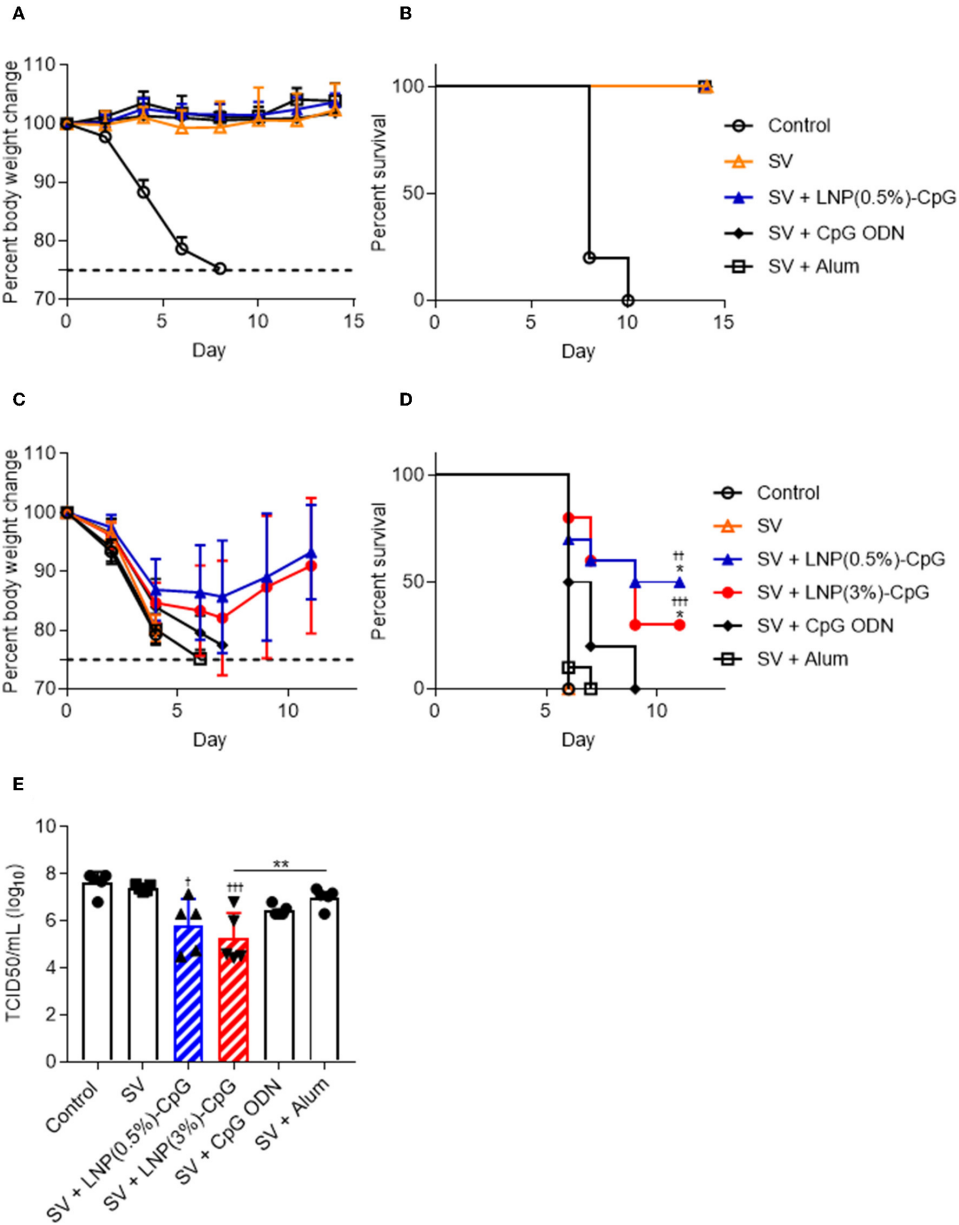
Preventive effects against influenza virus. Ten days after final immunization with SV from Cal7, mice were challenged with Cal7 or PR8. Percentages of initial body weights **(A,C)** and survival rates **(B,D)** were monitored after challenge with Cal7 **(A,B)** or PR8 **(C,D)**. Five days after PR8 challenge, virus titers in bronchoalveolar lavage fluid were measured **(E)**. **(A,B,E)** *n* = 5. **(C,D)** *n* = 9 or 10. Data are means ± SD. **(D)** ^††^*P* < 0.01, ^†††^*P* < 0.001 vs. group immunized with SV alone; *P < 0.05 vs. group immunized with SV plus CpG ODN, as indicated by comparing Kaplan-Meier curves using the log-rank test. **(E)** ^†^*P* < 0.05, ^†††^*P* < 0.001 vs. group immunized with SV alone; **P < 0.01 as indicated by Tukey's test.

After the final immunization, we challenged the immunized mice with Cal7 (Figures 6A,B) or PR8 (Figures 6C,D) and assessed their body weights (Figures 6A,C) and survival rates (Figures 6B,D). After Cal7 challenge, we did not observe any bodyweight loss or decrease in survival rate in any of the immunized mice, whereas unimmunized control mice showed rapid bodyweight loss, and all of them died within 10 days after challenge (Figures 6A,B). Unlike in the Cal7 challenge, much greater pathogenesis was observed after PR8 challenge in mice immunized with SV alone, or with SV plus CpG ODN, or with SV plus alum, as indicated by weight loss, and all of these mice died within 10 days after challenge (Figures 6C,D). In contrast, of the mice given SV plus either LNP-CpG, 30% [LNP(3%)-CpG] or 50% [LNP(0.5%)-CpG] survived, and they regained their body weights, although weight loss occurred initially (Figures 6C,D). On day 5 post-challenge, PR8 virus titers in the BALF of mice immunized with SV plus either LNP-CpG were significantly lower than that in mice immunized with SV alone, consistent with the body weight changes and survival rates (Figure 6E). These data indicated that LNP-CpG acted as an adjuvant to combat not only homologous-virus but also heterologous-virus challenge, thus broadening the protective spectrum of SV against influenza virus.

### LNP-CpG Has Strong Preventive Effects Against Heterosubtypic Influenza Virus Challenge After Tex50 SV Vaccination

**Figure 7:**
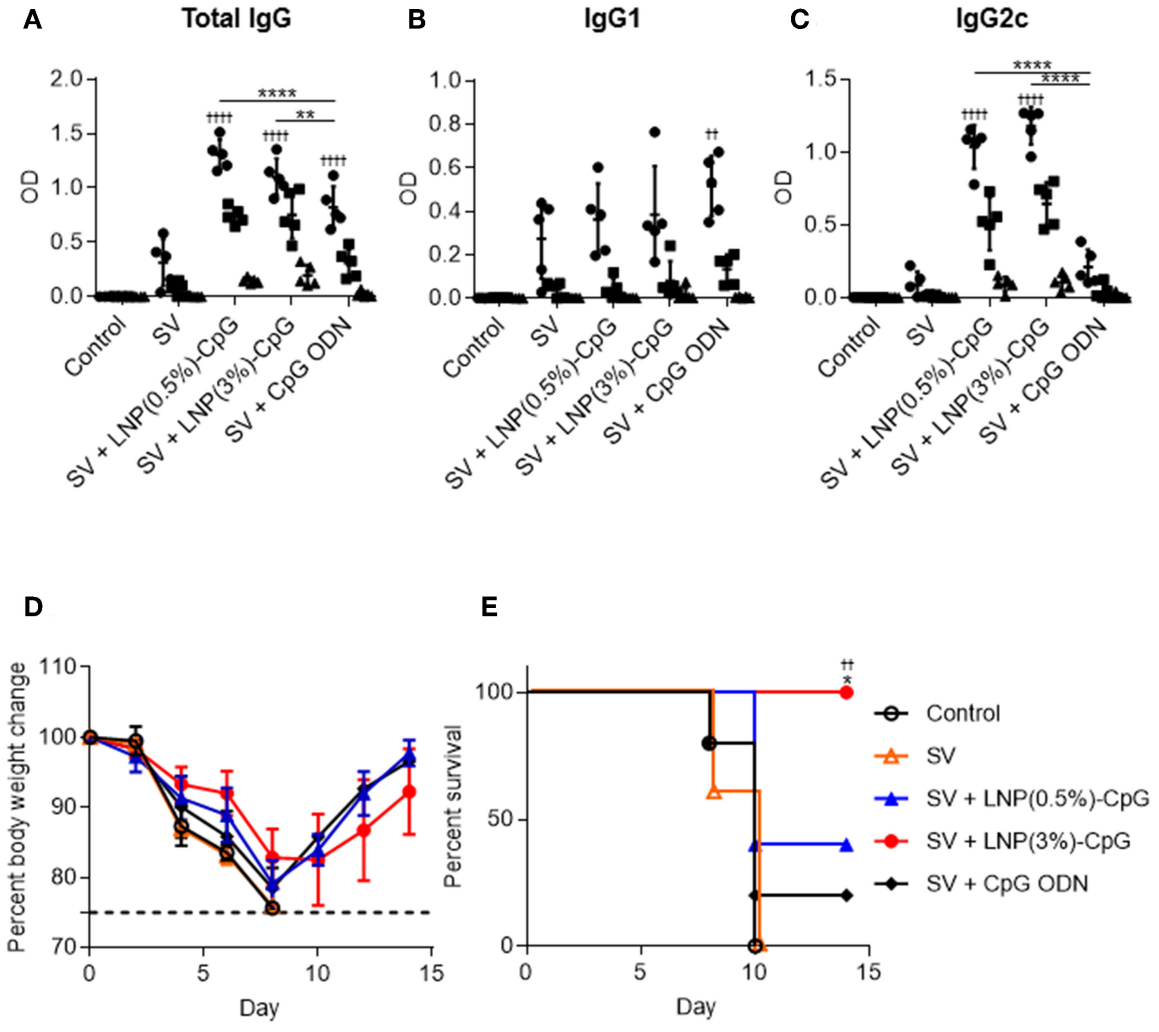
Preventive effects against heterosubtypic influenza virus. Mice were immunized subcutaneously with SV from Tex50 alone, SV plus CpG ODN, or SV plus either LNP-CpG. **(A–C)** Antibody responses. Levels of total IgG **(A)**, IgG1 **(B)**, and IgG2c **(C)** specific to SV from Tex50 in plasma were evaluated by using ELISA 7 days after final immunization. We used 4000- (•), 20,000- (■), and 100,000- (▴) fold diluted plasma samples. **(D,E)** Preventive effects against heterosubtypic Cal7. Ten days after final immunization, mice were challenged with Cal7. Percentages of initial body weights **(D)** and survival rates **(E)** were monitored after challenge with Cal7. **(A–E)** *n* = 5. Data are means ± SD. **(A–C)** Significant differences were analyzed only in the 4,000-fold-diluted plasma samples. ^††^*P* < 0.01, ^††††^*P* < 0.0001 vs. group immunized with SV alone; **P < 0.01, ****P < 0.0001 as indicated by Tukey's test. **(E)** ^††^*P* < 0.01 vs. group immunized with SV alone; *P < 0.05 vs. group immunized with SV plus CpG ODN, as indicated by comparing Kaplan–Meier curves through the log-rank test.

We examined the preventive effects of SV plus LNP-CpGs against heterosubtypic virus challenge. We used conventional seasonal SV from H3N2 Tex50 as an antigen and H1N1 PR8 as the challenge virus. Mice were immunized with SV from Tex50 plus either LNP-CpG (10 μg CpG ODN/mouse) or with SV plus CpG ODN (10 μg/mouse). Plasma levels of total IgG (Figure 7A), IgG1 (Figure 7B), and IgG2c (Figure 7C) antibodies specific to SV from Tex50 were analyzed by using ELISA after last immunization. Consistent with Figures 4A–C, the level of SV-specific total IgG in mice given SV plus either LNP-CpG was significantly higher than that in mice given SV plus CpG ODN (Figure 7A). SV plus either LNP-CpG induced significantly higher PR8-specific IgG2c levels than did SV plus CpG ODN (Figure 7C), whereas the level of SV-specific IgG1 in mice immunized with SV plus either LNP-CpG did not differ from that in mice immunized with SV alone or with SV plus CpG ODN (Figure 7B). After the final immunization, we challenged the immunized mice with Cal7 and assessed their body weights (Figure 7D) and survival rates (Figure 7E). All of the mice given SV plus LNP(3%)-CpG survived, and they regained their body weights, although weight loss occurred initially (Figures 7D,E). In contrast, mice immunized with SV plus CpG ODN or LNP(0.5%)-CpG did not show a significant improvement in survival rate compared with those given SV alone (Figure 7E). These data indicate that LNP(3%)-CpG acted as an adjuvant to combat not only heterologous-virus but also heterosubtypic-virus challenge.

## Discussion

Generally, PEG on the surface of LNPs reduces the positive charge of LNPs and suppresses not only the aggregation of each LNP and opsonization by biological proteins such as complement but also the interactions between LNPs and cells, indicating that PEG shielding of the LNPs influences their cellular uptake efficiency and biodistribution in the body (39–41). For example, greater PEG modification and modification with PEGs with longer chains inhibit the cellular uptake of LNPs, resulting in reduced cellular uptake of the molecules being delivered and inhibition of their desired functions, although these modifications inhibit opsonization, thus enhancing the stability of LNPs in the blood (39–41). Many authors have suggested that optimal modification of PEG with a molecular weight of 2000 is a better compromise between anti-opsonization and efficient delivery strategies (42). Therefore, we used two types of LNP modified with PEG at a molecular weight of 2000 in different ratios. We showed that each LNP-CpG improved the immune-stimulatory activity of CpG ODN in mouse DCs (Figure 1). This enhanced immune-stimulatory activity might result from an increase in the number of CpG ODNs taken up by each DC, because the CpG ODN is condensed into a space in the LNP-CpG. We showed that LNP(0.5%)-CpG had stronger immune-stimulatory activity (in terms of cytokine production and CD86 expression) than LNP(3%)-CpG in mouse DCs (Figure 1). Therefore, a greater number of LNP(0.5%)-CpG than LNP(3%)-CpG might have been taken up by the DCs, because the ratio of PEG modification was less in the former than in the latter. CpG ODN is localized to the endosomes or lysosomes after cellular internalization and binds to endosomal TLR9 (43, 44). In addition, by using TLR9 deficient mice, we recently showed that cytokine production by bone marrow cells in response to LNP-CpG is completely dependent on TLR9 (35). Therefore, we speculate that LNP-CpG might localize in the endosomes or lysosomes, releasing CpG ODN and inducing the activation of TLR9 signaling, although further study is required to clarify the exact cellular localization of LNP-CpG.

Compared with CpG-ODN, LNP(0.5%)-CpG enhanced the expression of CD80 and CD86 *in vivo*, in the same way as *in vitro* (Figure 2). In analyses of the biodistribution of particles *in vivo*, it has been suggested that small nanoparticles (diameter, 20–100 nm) are preferentially trafficked toward draining lymph nodes and are taken up by DCs in the lymph nodes, whereas smaller molecules tend to diffuse into the systemic circulation, with poor trafficking toward the draining lymph nodes (45). Therefore, we speculate that LNP(0.5%)-CpG and LNP(3%)-CpG might deliver CpG ODN into the draining lymph nodes more efficiently than CpG ODN alone. Furthermore, LNP(0.5%)-CpG enhanced the expression of CD80 and CD86 on pDCs (Figure 2). pDCs are a unique subset of DCs that can produce IFN-α and promote antiviral immune responses (46, 47). Recent studies have shown that pDCs express MHC class II molecules and co-stimulatory molecules and have the potential for antigen presentation to CD4^+^ T cells (46, 47). For example, Loschko et al. (48) showed that antigen delivery to pDCs by monoclonal antibody against bone marrow stromal antigen 2 with an adjuvant induced antigen presentation by the pDCs to CD4^+^ T cells, with increased expression of CD86 on the pDCs, resulting in the robust induction of CD4^+^ T-cell responses and antigen-specific antibody responses. Therefore, antigen presentation by pDCs might be indispensable in LNP-CpG-induced immune responses.

We found that the SV plus LNP-CpG—but not SV plus CpG ODN or SV plus alum—protected mice against not only a homologous strain (Cal7) but also an antigenically mismatched (heterologous) strain (PR8) (Figure 6). In addition, the antibodies induced by SV plus LNP-CpG did not have neutralizing activity against the heterologous strain (Figure 4), suggesting that the protection against heterologous PR8 challenge was not due to the production of neutralizing antibodies. Furthermore, SV plus LNP(3%)-CpG protected mice vaccinated with SV from Tex50 against a heterosubtypic strain (Cal7) (Figure 7), although it is important to examine why SV plus LNP(0.5%)-CpG did not show protective effects. It has been believed that neutralizing antibodies, which inhibit viral infection by hindering receptor binding on cells, are essential for protection against homologous influenza virus, but that these antibodies cannot protect against heterologous or heterosubtypic strains (49, 50). Conversely, recent studies have shown that non-neutralizing antibodies against influenza viruses contribute to cross-protection against both heterologous and heterosubtypic strains (49, 50). The cross-protection provided by non-neutralizing antibodies depends on the effector function of the Fc region via Fcγ receptor (FcγR) interaction, for example by antibody-dependent cellular cytotoxicity. Furthermore, mouse IgG2 has generally stronger antibody-dependent cellular cytotoxicity activity than IgG1, and it provides cross-protection more efficiently than does IgG1 because of its ability to interact with all activating FcγRs, such as FcγRI, FcγRIII, and FcγRIV (51–54). In fact, Van den Hoecke et al. (55) have shown that anti-influenza virus monoclonal IgG2 antibody has superior protective efficacy to monoclonal IgG1. Therefore, efficient induction of non-neutralizing IgG2 against influenza virus is a useful approach to the development of a universal influenza vaccine with cross-protection. We showed here that LNP-CpG improved the capacity of CpG ODN to induce antigen-specific IgG2 production; SV plus LNP-CpG induced PR8-specific IgG2 predominantly, whereas SV plus alum induced PR8-specific IgG1 predominantly (Figure 4). In addition, SV plus alum did not protect against heterologous influenza virus challenge (Figure 6), although the level of PR8-specific total IgG in mice immunized with SV plus alum was significantly greater than that in mice given LNP-CpG (Figure 4). Therefore, we speculate that non-neutralizing IgG2, not IgG1, induced by LNP-CpG against heterologous and heterosubtypic strains contributes to cross-protection. In contrast, after influenza infection, not only antibodies but also CD8^+^ T cells play crucial roles in cross-protection (56). However, Yamamoto et al. (57) showed that CD8^+^ T cells do not contribute to cross-protection in the immunization of mice with SV modified with CpG ODN, although SV modified with CpG ODN induces a strong cross-reactive CD8^+^ T cell response. The contribution of CD8^+^ T cells to cross-protection in the administration of LNP-CpG-adjuvanted vaccines needs to be explored.

In summary, we showed here that LNPs as CpG ODN delivery vehicles improved the immune-stimulatory activity of CpG ODN and that LNP-CpGs could broaden the protective spectrum of SV against influenza virus. We believe that LNP-CpGs can improve some influenza vaccines for pandemic influenza, as well as influenza vaccines for the elderly. We also believe that LNP-CpGs have the potential to open up new avenues for producing universal influenza vaccines with cross-protection and will help to develop novel adjuvant-delivery vehicles that could improve vaccine effectiveness.

## Data Availability Statement

The datasets generated for this study are available on request to the corresponding author.

## Ethics Statement

The animal study was reviewed and approved by Osaka University's institutional guidelines for the ethical treatment of animals (protocol number H26-11-0).

## Author Contributions

SS and YY designed the experiments, interpreted the results, and wrote the manuscript. SS, MS, AK, ST, LM, and DO performed the experiments and collected and analyzed the data. RS and TA provided technical support and conceptual advice. YY supervised the study.

### Conflict of Interest

TA and YY are employed by The Research Foundation for Microbial Diseases of Osaka University. RS and TA have filed a patent application related to the content of this manuscript. The remaining authors declare that the research was conducted in the absence of any commercial or financial relationships that could be construed as a potential conflict of interest.

## Abbreviations

Alum: aluminum salts
Cal7: H1N1 influenza A virus A/California/7/2009
cDC: conventional dendritic cell
CpG: unmethylated cytosine-phosphate-guanine
DC: dendritic cell
ELISA: enzyme-linked immunosolvent assay
FcγR: Fcγ receptor
HA: hemagglutinin
IFN: interferon
IL: interleukin
LNP: lipid nanoparticle
LNP-CpG: LNP containing CpG ODN
NA: neuraminidase
ODN: oligodeoxynucleotide
pDC: plasmacytoid dendritic cell
PEG: polyethylene glycol
PR8: H1N1 influenza A virus A/Puerto Rico/8/34
SV: (ether-treated hemagglutinin-antigen-enriched virion-free) split vaccine
Tex50: H3N2 influenza A virus A/Texas/50/2012
TLR9: Toll-like receptor 9.

## References

[1] Krammer F Palese P. Advances in the development of influenza virus vaccines. Nat Rev Drug Discov. (2015) 14:167–82. 10.1038/nrd4529 25722244

[2] Coughlan L Palese P. Overcoming barriers in the path to a universal influenza virus vaccine. Cell Host Microbe. (2018) 24:18–24. 10.1016/j.chom.2018.06.016 30001520

[3] Zimmerman RK Nowalk MP Chung J Jackson ML Jackson LA Petrie JG. 2014-2015 Influenza vaccine effectiveness in the United States by vaccine type. Clin Infect Dis. (2016) 63:1564–73. 10.1093/cid/ciw635 27702768 PMC5146719

[4] Krammer F. The human antibody response to influenza a virus infection and vaccination. Nat Rev Immunol. (2019) 19:383–97. 10.1038/s41577-019-0143-6 30837674

[5] Huber VC McKeon RM Brackin MN Miller LA Keating R Brown SA. Distinct contributions of vaccine-induced immunoglobulin G1 (IgG1) and IgG2a antibodies to protective immunity against influenza. Clin Vaccine Immunol. (2006) 13:981–90. 10.1128/CVI.00156-06 16960108 PMC1563571

[6] Clegg CH Roque R Van Hoeven N Perrone L Baldwin SL Rininger JA. Adjuvant solution for pandemic influenza vaccine production. Proc Natl Acad Sci USA. (2012) 109:17585–90. 10.1073/pnas.1207308109 23045649 PMC3491477

[7] McKee AS Marrack P. Old and new adjuvants. Curr Opin Immunol. (2017) 47:44–51. 10.1016/j.coi.2017.06.005 28734174 PMC5724967

[8] Hanagata N. Structure-dependent immunostimulatory effect of CpG oligodeoxynucleotides and their delivery system. Int J Nanomed. (2012) 7:2181–95. 10.2147/IJN.S30197 22619554 PMC3356174

[9] Shirota H Klinman DM. Recent progress concerning CpG DNA and its use as a vaccine adjuvant. Expert Rev Vaccines. (2014) 13:299–312. 10.1586/14760584.2014.863715 24308579 PMC6335645

[10] Marongiu L Gornati L Artuso I Zanoni I Granucci F. Below the surface: the inner lives of TLR4 and TLR9. J Leukoc Biol. (2019) 106:147–60. 10.1002/JLB.3MIR1218-483RR 30900780 PMC6597292

[11] Marschner A Rothenfusser S Hornung V Prell D Krug A Kerkmann M. CpG ODN enhance antigen-specific NKT cell activation via plasmacytoid dendritic cells. Eur J Immunol. (2005) 35:2347–57. 10.1002/eji.200425721 16025562

[12] Huber JP Farrar JD. Regulation of effector and memory T-cell functions by type I interferon. Immunology. (2011) 132:466–74. 10.1111/j.1365-2567.2011.03412.x 21320124 PMC3075500

[13] Verthelyi D Kenney RT Seder RA Gam AA Friedag B Klinman DM. CpG oligodeoxynucleotides as vaccine adjuvants in primates. J Immunol. (2002) 168:1659–63. 10.4049/jimmunol.168.4.1659 11823494

[14] Storni T Ruedl C Schwarz K Schwendener RA Renner WA Bachmann MF. Nonmethylated CG motifs packaged into virus-like particles induce protective cytotoxic T cell responses in the absence of systemic side effects. J Immunol. (2004) 172:1777–85. 10.4049/jimmunol.172.3.1777 14734761

[15] Senti G Johansen P Haug S Bull C Gottschaller C Muller P. Use of A-type CpG oligodeoxynucleotides as an adjuvant in allergen-specific immunotherapy in humans: a phase I/IIa clinical trial. Clin Exp Allergy. (2009) 39:562–70. 10.1111/j.1365-2222.2008.03191.x 19226280

[16] Speiser DE Schwarz K Baumgaertner P Manolova V Devevre E Sterry W. Memory and effector CD8 T-cell responses after nanoparticle vaccination of melanoma patients. J Immunother. (2010) 33:848–58. 10.1097/CJI.0b013e3181f1d614 20842051

[17] Goldinger SM Dummer R Baumgaertner P Mihic-Probst D Schwarz K Hammann-Haenni A. Nano-particle vaccination combined with TLR-7 and−9 ligands triggers memory and effector CD8(+) T-cell responses in melanoma patients. Eur J Immunol. (2012) 42:3049–61. 10.1002/eji.201142361 22806397 PMC3549564

[18] Junquera E Aicart E. Recent progress in gene therapy to deliver nucleic acids with multivalent cationic vectors. Adv Colloid Interface Sci. (2016) 233:161–75. 10.1016/j.cis.2015.07.003 26265376

[19] Cullis PR Hope MJ. Lipid nanoparticle systems for enabling gene therapies. Mol Ther. (2017) 25:1467–75. 10.1016/j.ymthe.2017.03.013 28412170 PMC5498813

[20] Takahashi H Misato K Aoshi T Yamamoto Y Kubota Y Wu X. Carbonate apatite nanoparticles act as potent vaccine adjuvant delivery vehicles by enhancing cytokine production induced by encapsulated cytosine-phosphate-guanine oligodeoxynucleotides. Front Immunol. (2018) 9:783. 10.3389/fimmu.2018.00783 29720976 PMC5916113

[21] Kulkarni JA Cullis PR van der Meel R. Lipid nanoparticles enabling gene therapies: from concepts to clinical utility. Nucleic Acid Ther. (2018) 28:146–57. 10.1089/nat.2018.0721 29683383

[22] Belliveau NM Huft J Lin PJ Chen S Leung AK Leaver TJ. Microfluidic synthesis of highly potent limit-size lipid nanoparticles for *in vivo* delivery of siRNA. Mol Ther Nucleic Acids. (2012) 1:e37. 10.1038/mtna.2012.28 23344179 PMC3442367

[23] Zhigaltsev IV Belliveau N Hafez I Leung AK Huft J Hansen C. Bottom-up design and synthesis of limit size lipid nanoparticle systems with aqueous and triglyceride cores using millisecond microfluidic mixing. Langmuir. (2012) 28:3633–40. 10.1021/la204833h 22268499

[24] Barros SA Gollob JA. Safety profile of RNAi nanomedicines. Adv Drug Deliv Rev. (2012) 64:1730–7. 10.1016/j.addr.2012.06.007 22732527

[25] Coelho T Adams D Silva A Lozeron P Hawkins PN Mant T. Safety and efficacy of RNAi therapy for transthyretin amyloidosis. N Engl J Med. (2013) 369:819–29. 10.1056/NEJMoa1208760 23984729

[26] DeRosa F Guild B Karve S Smith L Love K Dorkin JR. Therapeutic efficacy in a hemophilia B model using a biosynthetic mRNA liver depot system. Gene Ther. (2016) 23:699–707. 10.1038/gt.2016.46 27356951 PMC5059749

[27] Pardi N Secreto AJ Shan X Debonera F Glover J Yi Y. Administration of nucleoside-modified mRNA encoding broadly neutralizing antibody protects humanized mice from HIV-1 challenge. Nat Commun. (2017) 8:14630. 10.1038/ncomms14630 28251988 PMC5337964

[28] Ramishetti S Kedmi R Goldsmith M Leonard F Sprague AG Godin B. Systemic gene silencing in primary T lymphocytes using targeted lipid nanoparticles. ACS Nano. (2015) 9:6706–16. 10.1021/acsnano.5b02796 26042619

[29] Geall AJ Verma A Otten GR Shaw CA Hekele A Banerjee K. Nonviral delivery of self-amplifying RNA vaccines. Proc Natl Acad Sci USA. (2012) 109:14604–9. 10.1073/pnas.1209367109 22908294 PMC3437863

[30] Pardi N Hogan MJ Pelc RS Muramatsu H Andersen H DeMaso CR. Zika virus protection by a single low-dose nucleoside-modified mRNA vaccination. Nature. (2017) 543:248–51. 10.1038/nature21428 28151488 PMC5344708

[31] Richner JM Himansu S Dowd KA Butler SL Salazar V Fox JM. Modified mRNA vaccines protect against zika virus infection. Cell. (2017) 168:1114–25.e10. 10.1016/j.cell.2017.02.017 28222903 PMC5388441

[32] Bahl K Senn JJ Yuzhakov O Bulychev A Brito LA Hassett KJ. Preclinical and clinical demonstration of immunogenicity by mRNA vaccines against H10N8 and H7N9 influenza viruses. Mol Ther. (2017) 25:1316–27. 10.1016/j.ymthe.2017.03.035 28457665 PMC5475249

[33] Liang F Lindgren G Lin A Thompson EA Ols S Rohss J. Efficient targeting and activation of antigen-presenting cells *in vivo* after modified mRNA vaccine administration in rhesus macaques. Mol Ther. (2017) 25:2635–47. 10.1016/j.ymthe.2017.08.006 28958578 PMC5768558

[34] John S Yuzhakov O Woods A Deterling J Hassett K Shaw CA. Multi-antigenic human cytomegalovirus mRNA vaccines that elicit potent humoral and cell-mediated immunity. Vaccine. (2018) 36:1689–99. 10.1016/j.vaccine.2018.01.029 29456015

[35] Munakata L Tanimoto Y Osa A Meng J Haseda Y Naito Y. Lipid nanoparticles of Type-A CpG D35 suppress tumor growth by changing tumor immune-microenvironment and activate CD8 T cells in mice. J Control Release. (2019) 313:106–19. 10.1016/j.jconrel.2019.09.011 31629036

[36] Wei CJ Xu L Kong WP Shi W Canis K Stevens J. Comparative efficacy of neutralizing antibodies elicited by recombinant hemagglutinin proteins from avian H5N1 influenza virus. J Virol. (2008) 82:6200–8. 10.1128/JVI.00187-08 18417563 PMC2447076

[37] Prevato M Ferlenghi I Bonci A Uematsu Y Anselmi G Giusti F. Expression and characterization of recombinant, tetrameric and enzymatically active influenza neuraminidase for the setup of an enzyme-linked lectin-based assay. PLoS ONE. (2015) 10:e0135474. 10.1371/journal.pone.0135474 26280677 PMC4539205

[38] Tamura S Samegai Y Kurata H Nagamine T Aizawa C Kurata T. Protection against influenza virus infection by vaccine inoculated intranasally with cholera toxin B subunit. Vaccine. (1988) 6:409–13. 10.1016/0264-410X(88)90140-5 2848377

[39] Kumar V Qin J Jiang Y Duncan RG Brigham B Fishman S. Shielding of lipid nanoparticles for sirna delivery: impact on physicochemical properties, cytokine induction, and efficacy. Mol Ther Nucleic Acids. (2014) 3:e210. 10.1038/mtna.2014.61 25405467 PMC4459547

[40] Chen S Tam YY Lin PJ Leung AK Tam YK Cullis PR. Development of lipid nanoparticle formulations of siRNA for hepatocyte gene silencing following subcutaneous administration. J Control Release. (2014) 196:106–12. 10.1016/j.jconrel.2014.09.025 25285610

[41] Zatsepin TS Kotelevtsev YV Koteliansky V. Lipid nanoparticles for targeted siRNA delivery - going from bench to bedside. Int J Nanomedicine. (2016) 11:3077–86. 10.2147/IJN.S106625 27462152 PMC4939975

[42] Pozzi D Colapicchioni V Caracciolo G Piovesana S Capriotti AL Palchetti S. Effect of polyethyleneglycol (PEG) chain length on the bio-nano-interactions between PEGylated lipid nanoparticles and biological fluids: from nanostructure to uptake in cancer cells. Nanoscale. (2014) 6:2782–92. 10.1039/c3nr05559k 24463404

[43] Honda K Ohba Y Yanai H Negishi H Mizutani T Takaoka A. Spatiotemporal regulation of MyD88-IRF-7 signalling for robust type-I interferon induction. Nature. (2005) 434:1035–40. 10.1038/nature03547 15815647

[44] Sasai M Linehan MM Iwasaki A. Bifurcation of Toll-like receptor 9 signaling by adaptor protein 3. Science. (2010) 329:1530–4. 10.1126/science.1187029 20847273 PMC3063333

[45] Moyer TJ Zmolek AC Irvine DJ. Beyond antigens and adjuvants: formulating future vaccines. J Clin Invest. (2016) 126:799–808. 10.1172/JCI81083 26928033 PMC4767337

[46] Swiecki M Colonna M. The multifaceted biology of plasmacytoid dendritic cells. Nat Rev Immunol. (2015) 15:471–85. 10.1038/nri3865 26160613 PMC4808588

[47] Reizis B. Plasmacytoid dendritic cells: development, regulation, and function. Immunity. (2019) 50:37–50. 10.1016/j.immuni.2018.12.027 30650380 PMC6342491

[48] Loschko J Schlitzer A Dudziak D Drexler I Sandholzer N Bourquin C. Antigen delivery to plasmacytoid dendritic cells via BST2 induces protective T cell-mediated immunity. J Immunol. (2011) 186:6718–25. 10.4049/jimmunol.1004029 21555533

[49] Neu KE Henry Dunand CJ Wilson PC. Heads, stalks and everything else: how can antibodies eradicate influenza as a human disease? Curr Opin Immunol. (2016) 42:48–55. 10.1016/j.coi.2016.05.012 27268395 PMC5086271

[50] Jegaskanda S Vanderven HA Wheatley AK Kent SJ. Fc or not Fc; that is the question: antibody Fc-receptor interactions are key to universal influenza vaccine design. Hum Vaccin Immunother. (2017) 13:1–9. 10.1080/21645515.2017.1290018 28332900 PMC5489290

[51] Markine-Goriaynoff D Coutelier JP. Increased efficacy of the immunoglobulin G2a subclass in antibody-mediated protection against lactate dehydrogenase-elevating virus-induced polioencephalomyelitis revealed with switch mutants. J Virol. (2002) 76:432–5. 10.1128/JVI.76.1.432-435.2002 11739710 PMC135718

[52] Schmitz N Beerli RR Bauer M Jegerlehner A Dietmeier K Maudrich M. Universal vaccine against influenza virus: linking TLR signaling to anti-viral protection. Eur J Immunol. (2012) 42:863–9. 10.1002/eji.201041225 22531913

[53] El Bakkouri K Descamps F De Filette M Smet A Festjens E Birkett A. Universal vaccine based on ectodomain of matrix protein 2 of influenza A: Fc receptors and alveolar macrophages mediate protection. J Immunol. (2011) 186:1022–31. 10.4049/jimmunol.0902147 21169548

[54] Beers SA Glennie MJ White AL. Influence of immunoglobulin isotype on therapeutic antibody function. Blood. (2016) 127:1097–101. 10.1182/blood-2015-09-625343 26764357 PMC4797141

[55] Van den Hoecke S Ehrhardt K Kolpe A El Bakkouri K Deng L Grootaert H. Hierarchical and redundant roles of activating fcgammars in protection against influenza disease by M2e-specific IgG1 and IgG2a antibodies. J Virol. (2017) 91:e02500–16. 10.1128/JVI.02500-16 28077656 PMC5355615

[56] Laidlaw BJ Decman V Ali MA Abt MC Wolf AI Monticelli LA. Cooperativity between CD8+ T cells, non-neutralizing antibodies, and alveolar macrophages is important for heterosubtypic influenza virus immunity. PLoS Pathog. (2013) 9:e1003207. 10.1371/journal.ppat.1003207 23516357 PMC3597515

[57] Yamamoto T Masuta Y Momota M Kanekiyo M Kanuma T Takahama S. A unique nanoparticulate TLR9 agonist enables a HA split vaccine to confer FcgammaR-mediated protection against heterologous lethal influenza virus infection. Int Immunol. (2019) 31:81–90. 10.1093/intimm/dxy069 30535055 PMC6599278

